# Bitumen Aging—Laboratory Simulation Methods Used in Practice and Selected Directions of Research on New Methods

**DOI:** 10.3390/ma16020853

**Published:** 2023-01-16

**Authors:** Paweł Czajkowski, Andrzej Przyjazny, Grzegorz Boczkaj

**Affiliations:** 1 Department of Sanitary Engineering, Faculty of Civil and Environmental Engineering, Gdańsk University of Technology, 80-233 Gdansk, Poland; 2 Rafineria Gdańska, Elbląska 135 Str., 80-718 Gdansk, Poland; 3 Department of Natural Sciences, Kettering University, 1700 University Avenue, Flint, MI 48504, USA; 4 EkoTech Center, Gdansk University of Technology, G. Narutowicza Str. 11/12, 80-233 Gdansk, Poland

**Keywords:** bitumen, binder, laboratory aging methods, roads, chemical degradation, oxidation

## Abstract

Changes in the properties of bitumen binders that occur as a result of aging have a huge impact on the durability of products produced from them. In particular, asphalt pavements, which constitute the most common use of petroleum bitumen, are susceptible to damage resulting from the increasing stiffness of the bitumen during its life cycle. Increased stiffness of asphalt pavements reduces the pavement resistance to low-temperature cracks and fatigue cracks, ultimately leading to the loss of their functional properties and the need for road repair. The rate of changes in bitumen properties is influenced by many factors, the most important of which are environmental conditions, technological parameters of binder processing, and physicochemical properties. The greatest impact on minimizing the adverse effect of aging is the use of bitumen suitably resistant to aging, and changing the technological parameters of its application. This article reviews the literature and standardized test methods of bitumen aging, with a focus on the methods that are most often used in practice, to evaluate the suitability of bitumen for use in road construction. The presented methods are limited to aging simulation. This mini-review presents the most important stages of aging procedures, their advantages and limitations, as identified by the authors of this publication for different types of bitumen. Moreover, the most important directions of developments in the field of new laboratory aging tests are highlighted. The suggestions are based on the industrial practice of the authors of this review, taking into account identified demands for quality control in the industry.

## 1. Introduction

Petroleum bitumen is a well-known product for the construction of asphalt pavements. In Poland, at the end of 2019, over 80% of the roads in the highest traffic category were constructed with bitumen binder [1,2]. On the other hand, in Europe, this share amounts to about 90%, although it is difficult to verify because relevant statistics are not available. In the United States, according to an analysis based on 2014 data, about 94% of roads have asphalt surfaces [3]. Currently, both in Poland and throughout Europe, a wide range of bitumen for road construction is available, ranging from traditional petroleum bitumen produced during the processing of crude oil, through to polymer modified bitumen, and ending with special bitumen containing additives improving the functional properties of the product. Depending on the end use of a given road, the correct selection of bitumen and type of asphalt mix for the pavement structure largely determines its durability.

The properties of bitumen are variable during its life cycle. Bitumen is susceptible to aging, which depends on the type of crude oil it comes from, the production technology, and the technology of the final product application. In addition, bitumen aging continues over the entire life cycle of the end product. In simple terms, this process changes the chemical composition of the binder and, consequently, the physicochemical parameters, resulting in an increased hardness of bitumen, resistance to flow, as well as brittleness. Considering the durability of the road surface, the changes that occur during the aging process are unfavorable. Although it would seem that the hardness of the binder, which increases with time, allows for increased resistance to road rutting, in the overall balance, changes in bitumen adversely affect the life of the pavement, causing its significant reduction. The changes taking place in the bitumen during the use of the road contribute to the deterioration of low-temperature properties and reduction in the fatigue resistance of the asphalt pavement, which determine the durability and suitability of the pavement for carrying traffic loads.

The phenomenon of changes in the properties of bitumen was described, among others, in work from the 1960s [4], where it was proved that the changes occur most intensively in the outer layer of the road, the so-called wearing course. Bitumen binder parameters currently characterized by a dynamic shear rheometer (DSR) include the complex shear modulus (G*) and the phase angle (δ). With the aging of the bitumen pavement, higher G* and lower δ are recorded in the binders recovered from the upper layers compared with the bitumen used in the binder and subbase layers [5]. The binder undergoes chemical and physical changes under the influence of cycles of temperature changes, contact with moisture and air—especially oxygen, human-generated pollution, as well as UV radiation, and transferred loads. The deeper layers of the surface, i.e., the binder layer and subbase, have limited contact with factors accelerating the aging process compared with the wearing course.

When the road requires rehabilitation, the available road technologies make it possible to recover the previously used construction material from the pavement, which is called reclaimed asphalt. It consists of aggregate surrounded by asphalt mastic, i.e., a binder with a mineral filler. After several decades of pavement use, taking into account inflation, the materials used, such as bitumen or aggregate, have a higher value during pavement rehabilitation than during road construction. Therefore, one of the aspects driving the development of recycling is the financial benefit. In addition to the aspects resulting from technical or business requirements, the use of sustainable development strategies, particularly those limiting the impact on the natural environment, are becoming more and more popular in organizations. Therefore, the future of solutions based on the reuse of recovered asphalt mix for the construction of new roads looks bright.

## 2. Chemical Structure of Bitumen and Effects of Hardening of Binder on the Chemical Composition

The chemical structure of petroleum bitumen is determined by the properties of crude oil and the production technology used to achieve the final parameters of the bitumen binder. Bitumen is usually one of the highest boiling, high viscosity fractions obtained in the process of refining crude oil. Only the residues after extraction with propane or butane of vacuum fractions and petroleum coke are characterized by a higher concentration of compounds with the highest (relative to crude oil components) boiling point and the largest number of carbon atoms.

Detailed chemical analysis of bitumen is practically impossible to perform, mainly because of the complex structure of chemical compounds and the number of isomers present in the bitumen and their variability, depending on the variability of the crude oil composition. Therefore, in the oil and construction industry, there is no bitumen specification based on a chemical analysis of its composition, and there are no known correlations between the chemical properties of bitumen binders and the performance parameters of bitumen, which can be used universally, i.e., regardless of the origin of crude oil and bitumen production technology. Application of methods of instrumental analysis such as visible light (VIS) and near ultraviolet (UV) spectroscopy, Fourier transform infrared spectroscopy (FT-IR), nuclear magnetic resonance spectroscopy (NMR) combined with mass spectrometry (MS), however, due to the complex and variable composition of bitumen, do not allow a complete characterization of the chemical composition of bitumen binders [6]. Nevertheless, the analysis of the composition enables the explanation of changes in properties that occur, for example, with aging. In addition, chemical analysis allows for the identification of changes caused by chemical modification of bitumen, the assessment of the effectiveness of such modification, or the durability of the modification effects during the life cycle of the asphalt pavement (durability and stability of bitumen modification). However, it should be borne in mind that in most cases the identified chemical changes are not consistent with changes in the rheological properties of bitumen [7].

Due to the complexity of its structure, in order to determine the approximate chemical structure of bitumen, both elemental analysis (C, H, N, S, O) and the group composition analysis, the so-called SARA (the name comes from the first letters of four hydrocarbon groups, i.e., *Saturates Aromatics*, *Resins*, *Asphaltenes*) are still used today. The group composition test divides the binder sample into hydrocarbon compounds that differ in polarity, i.e., from the least polar saturates, through aromatics, resins and asphaltenes.

Elemental analysis is an analytical technique that is used in a wide range of applications and industries. The most commonly used technique for analyzing the elemental composition, CHNSO, is the procedure involving the combustion of a sample and then analyzing the gaseous combustion products (CO_2_, CO, H_2_O, NO_x_, SO_2_, etc.). The combustion products are determined by gas chromatography, by which the percentages of C, H, N, and S can be determined. The oxygen content is determined in the second stage of determination by pyrolysis [8].

**Figure 1:**
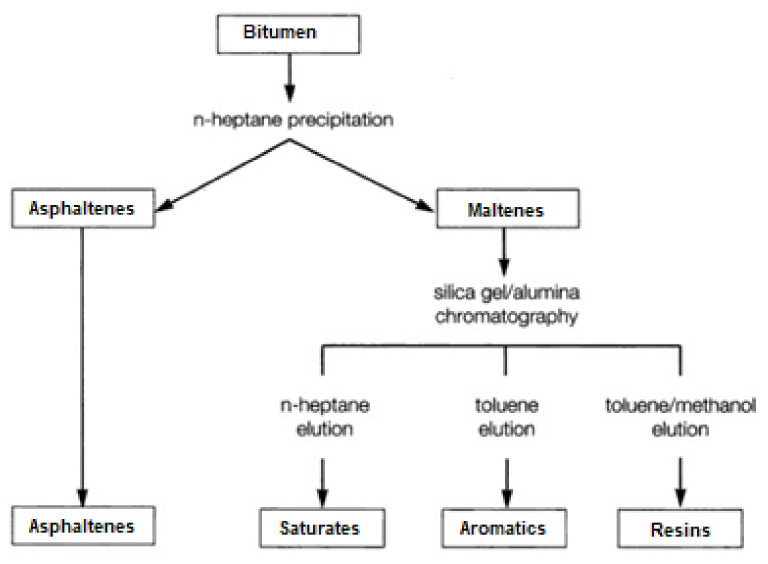
Schematic for the group separation of chemical constituents in bitumen.

The separation of bitumen into group components is most often carried out by following ASTM-D4124 [9]. The method involves separation into four defined fractions of petroleum hydrocarbons, i.e., saturated, naphthenic-aromatic, polar aromatic (resins), and insoluble in *n*-heptane (or isooctane) asphaltenes. The standardized method was developed and is evolving based on the analytical procedure used by Corbett and Swarbrick [10]. The principle of dividing into individual groups is shown in Figure 1.

**Figure 2:**
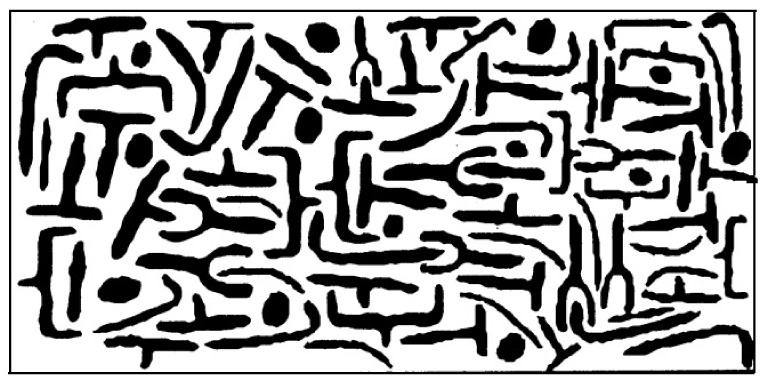
Model of the chemical structure of bitumen developed in the SHRP program [13]. The polar hydrocarbon systems are shown in black, while the white denotes the area representing nonpolar hydrocarbons.

In terms of its chemical composition, the bitumen binder produced by the processing of crude oil is a mixture of high-boiling hydrocarbons, most of which have a boiling point above 525 °C. The elemental composition by weight of the binder used in road construction is usually 80–88% carbon, 8–12% hydrogen, 0–9% sulfur, 0–2% nitrogen, and 0–2% oxygen. The remaining elements, such as vanadium, nickel, and magnesium, are within a maximum range of several hundred ppm [11]. In terms of its chemical structure, the binder has been described in the literature using colloidal or micellar models, and recently using the so-called microstructural model. As part of the Strategic Highway Research Program (SHRP), which is described in more detail in the sections on aging and bitumen specification, a chemical model of bitumen was also developed which, according to the authors, better explains the viscoelastic behavior of the binder than, for example, the micellar model. In the micellar model, the molecules with the highest molar mass—asphaltenes—form agglomerates that are dispersed in a matrix of maltenes consisting of resins, aromatic and saturated hydrocarbons. The microstructural model assumes that bitumen is a monophasic mixture of very complex hydrocarbons of polar (asphaltenes and resins) and nonpolar (aromatic and saturated hydrocarbons) orientation. In this system, there are no micelles, networks, or “floating” islands of asphaltenes. However, the fact is that a mixture of fractions with different polarities and molar masses interact with each other to form chemical complexes. Between nonpolar compounds, polar substances form these complexes as a result of weak electrostatic attractions, hydrogen bonds, and, to a lesser extent, through π-π bonds of aromatic hydrocarbon rings or van der Waals forces in the case of long-chain hydrocarbons [12]. Intermolecular bonds can be broken by temperature increase or shear forces; thus, the bitumen binder after the destruction of these assemblies, e.g., as a result of increasing the temperature, behaves as a Newtonian liquid, and when it is supercooled, the bonds re-form, but in a slightly different configuration. An illustration of the microstructural model is presented in Figure 2.

Bitumen, with the desired properties and chemical composition, is a material characterized by the appropriate proportion of polar and nonpolar fractions. The share is difficult to define unequivocally as it depends on the source of origin and the production technology of the bitumen binder. However, this specific balance in the chemical composition is a balance of substances that ensures the desired resistance of the material to damage. The low-temperature stability is determined mainly by the composition of the nonpolar fraction, while the fatigue cracking resistance and high-temperature deformation resistance depend on the share of polar fractions. The higher the share of polar fractions, the higher the properties of bitumen at a higher temperature, while the resistance to damage at intermediate temperatures decreases.

**Figure 3:**
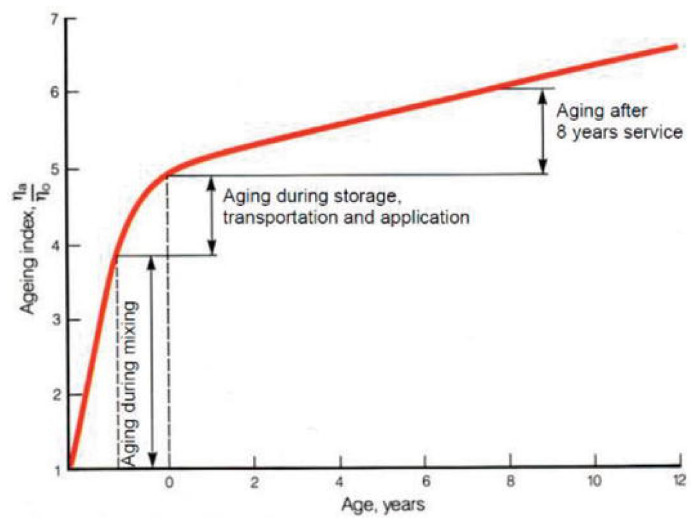
Aging effects on binder properties represented by the viscosity ratio index during the life of the bitumen [14].

Aging should be defined as a change in the properties of the bitumen binder as a result of chemical and physical changes that take place under the influence of environmental conditions such as oxygen, temperature, moisture, civilization pollution present in the air, and radiation (especially in the UV range). When used in road construction, there is an additional impact of vehicle traffic and the influence of components used for the production of asphalt mix. The oxidations by oxygen as well as evaporation and adsorption of fractions in the mineral components of asphalt mix, taking place at high processing temperatures, have the greatest impact on the rate and scope of changes in bitumen. The most intensive changes registered during the life cycle used in road construction (Figure 3) are related to the asphalt mix production process [14]. The subsequent stages associated with transport and asphalt mix incorporation are of lesser importance, and due to the decreasing asphalt mix temperature compared with the previous stages, the aging process of the binder strongly slows down at the stage of usage of the asphalt pavement. On the other hand, the use of an asphalt road is designed for at least 30 years, and more often for about 50 years, so due to the duration of the road’s operation, the long-term aging stage also plays a significant role in the durability of roads.

On the basis of infrared spectroscopy (FTIR), it is possible to estimate the changes that occur in bitumen binder, using the identification of bitumen functional groups that undergo oxidation during the life cycle. With the infrared spectra of the bitumen before and after aging and using the relationship between the peak area of the group undergoing chemical changes as a result of aging to the peak area, e.g., of aliphatic groups (-CH_2_CH_3_ or -CH_3_), appropriate aging indices can be determined. The relationship for the calculation of the C=O index is given by Formula (1):

ICO = Ax/Ay,
(1)

where: Ax—peak area at wavenumber 1666–1746 cm^−1^, characteristic for vibrations of the -C=O bond, and Ay is the reference peak area of the aliphatic group at 1319–1520 cm^−1^.

The studies based on the recorded CO and SO indices have shown that the identified changes in the chemical composition of bitumen after short-term aging are insignificant, while changes indicating a greater intensity of oxidation are detected only after long-term aging [15]. However, e.g., in modified bitumen, polymer degradation is detected, which is indicated by the SBS index, an aging index suitable for the modifier of the SBS type (styrene-butadiene-styrene block polymer).

On the other hand, based on the results of determination of the SARA group composition presented, e.g., in [16], it can be concluded that in the aged bitumen (designated as OA) the content of aromatic compounds is reduced compared with the proportion of this fraction in unaged bitumen (designated as VA), while the content of resins and asphaltenes increases, respectively. The above changes are a result of the evaporation of light components and the conversion of other hydrocarbons into more complex ones with a higher molar mass. It should be noted that in the mixtures tested in this paper, unaged and aged bitumen, where selected rejuvenating agents (substances regenerating the properties of the product) were additionally used, it has been shown that it is possible to achieve similar shares of individual groups of hydrocarbons, which are characteristic for bitumen before aging. 

Based on the study of the group composition, it is possible to determine the so-called colloidal instability index (CII), which is determined according to Formula (2). A higher index (CII) means lower stability of asphaltenes in the system. As a result, bitumen structure tends to gel—creating more and more complex aggregates of polar compounds, which may accelerate the asphalt pavement degradation process.

(2) $$CII=\frac{saturates+asphaltenes}{aromatics+resins}$$

Based on the same studies [16], it was shown that aged bitumen binder has the highest CII index, while bitumen resulting from mixing aged bitumen binder and a selected rejuvenating additive showed a lower CII value. This points to the fact that, for example, properly selected rejuvenating agents can restore the balance of colloidal stability of bitumen that has been subjected to excessively high aging, thus, effectively rebuilding the colloidal structure of such a mixture. The reference values for the CII index were not found; in this case, this index should be considered tentative and helpful for the comparative analysis of products. In the literature, the CII values reported most often are for crude oil, where it is assumed that when CII is <0.7, the crude is stable, and when the CII value is >0.9, crude oil can be considered unstable, and during its storage and processing the precipitation of asphaltenes may take place [17].

However, it should be noted that when assessing the technical characteristics of a product, one should always take into account a set of parameters covering a wide range of properties, which should preferably be correlated with the performance properties. The purpose of this approach is to exclude the use of single selected indicators or parameters, so as not to exclude a product, such as bitumen, that historically showed high durability in asphalt pavement.

## 3. Laboratory Bitumen Aging Methods

### 3.1. Short-Term Aging

Due to the relatively small changes in bitumen properties during service aging, the assessment of bitumen resistance to aging would be time-consuming without the developed and implemented laboratory methods based on the accelerating bitumen aging phenomenon. Currently, there are both standardized methods of short-term and long-term aging of bitumen as well as new solutions being developed in research centers.

The short-term aging simulation is intended to represent the conditions under which bitumen is hot-processed (i.e., >150 °C) into hot mix asphalt (HMA) until it is incorporated into the pavement. The greatest changes in asphalt among the subsequent technological stages of HMA production occur in the drum of the asphalt mix production plant. Bitumen, stored in a tank at a temperature of 160–180 °C, is introduced into the drum, where it contacts the surface of the hot aggregate. The standard production temperature is 170 °C. Then HMA goes to storage silos and thereafter is transported by car to the construction site where HMA is poured and compacted at a temperature below 150 °C. The aging changes of the binder are influenced by the high temperature after the stage of contact with the aggregate; the storage and transport time during which the HMA is kept at about 170 °C and is in contact with air becomes crucial.

**Figure 4:**
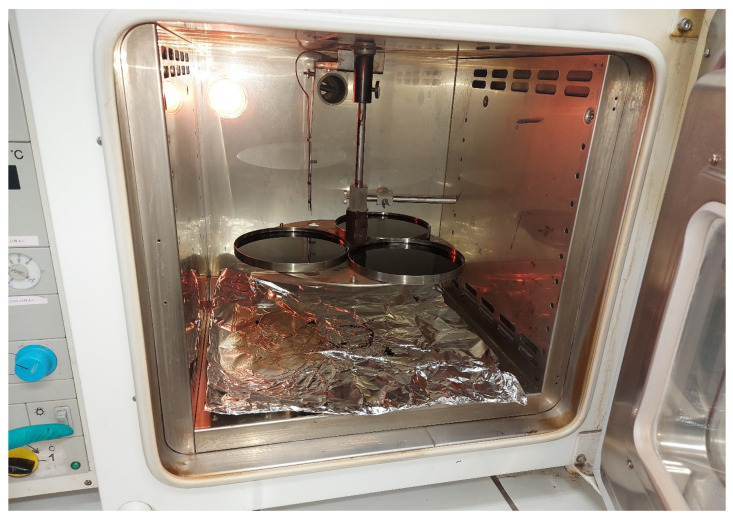
Photograph of a set of pans and an oven for aging bitumen by the TFOT method.

The first common method used to assess changes in bitumen properties after short-term aging is the TFOT (*Thin Film Oven Test*) method. TFOT aging simulation was implemented by Lewis and Welborn in 1940 [18]. It was standardized, among others, by CEN TC 336 as the European standard EN 12607-2 [19]. The determination consists of placing 50 mL of bitumen on a pan with a diameter of 140 mm and a height of 9.5 mm to obtain a thin layer of the binder with a thickness of about 3.2 mm. Next, the samples in the pan are placed on a rotating shelf in an oven for 5 h at 163 °C under atmospheric pressure. The bitumen sample is exposed to temperature and air. Figure 4 shows a picture of the TFOT device.

The TFOT method is used to compare the properties of bitumen in terms of resistance to weight loss due to the evaporation of the lowest boiling hydrocarbons and curing due to heating and contact with atmospheric oxygen.

As a result of this test procedure, aging takes place most intensively on the bitumen binder surface, which is its major drawback (beyond the test time), which in extreme cases can lead to the formation of a hard layer, which can prevent uniform aging. Currently, the TFOT method is used only for soft road bitumen, such as V6000 or V12000, which are classified by determining their viscosity at 60 °C, as the value of penetration of these binders is >400 (given in units of penetration, i.e., 0.1 mm).

**Figure 5:**
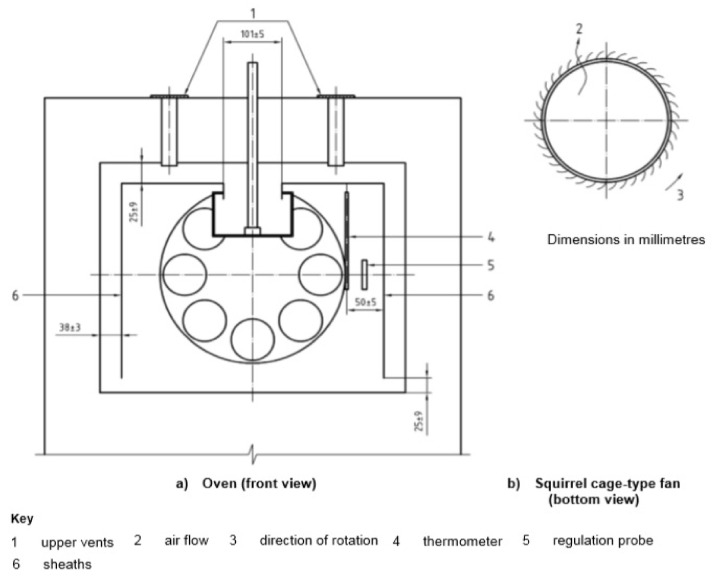
Oven and squirrel cage-type fan for aging by the RTFOT method (EN 12607-1:2014).

The evolution of the thin film aging test is the RTFOT (*Rolling Thin Film Oven Test*), a method developed by the California Division of Highways [20]. The aging procedure is described, among others, in the EN 12607-1 standard [21]. Figure 5 shows a schematic of the RTFOT. Currently, RTFOT is used to simulate the short-term aging of the most widely used road and polymer-modified bitumen. The change primarily involves the container in which the sample is placed, from a pan to a glass vessel, which is placed in a special holder. During the RTFOT test, the glass vessel is rotated and the binder can flow when exposed to temperature and air. Due to the smaller sample size, approximately 35 g, the rotating layer of bitumen needs a shorter time to reflect the changes occurring in the process of technological aging in the binder. During the test, the samples are rotated 15 times per minute and the airflow is set at 4000 mL/min. The RTFOT aging temperature is identical to that of the TFOT method, i.e., 163 °C. On the other hand, the aging procedure time is reduced from 5 h to 75 min according to the European specification, and 85 min in the case of using the AASHTO T240 [22] or ASTM D2872 [23] standards, i.e., the laboratory procedures used, among others, in the U.S.A.

RTFOT aging is the most widely used test method in the world for simulating the short-term aging of bitumen. In the case of testing the resistance to aging of paving grade bitumen, the RTFOT method is sufficient to assess the resistance to aging [24], although the temperature of 163 °C was selected only in terms of the hot technology and does not reflect changes during technological aging in a less energy-consuming warm technology warm mix asphalt (WMA).

The greatest difficulties arise when simulating the aging of high-viscosity bitumen, especially those modified with polymers. Bitumen does not rotate in the vessels, and, consequently, the thin film is not renewed. Thus, as in the TFOT method, aging occurs on the surface, and the air is not in contact with the entire mass of the sample. Various modifications of the RTFOT test have tried to eliminate this problem, e.g., metal rods were introduced into glass containers [25] to generate shear forces and thin layer of even, highly viscous, binders. However, this modification was not accepted by the industry as an effective solution to the problem. Moreover, it is recommended in the EN 14023:2010 standard, specifying polymer modified bitumen (PMB), to increase the RTFOT temperature to 180 °C for hard and highly viscous PMB bitumen. Nevertheless, the temperature increase in the RFTOF method does not fully eliminate the phenomenon of no-rotation of high-viscosity bitumen, thus, contributing to an increase in the aging effect of PMBs aged in such a way, which may lead to an incorrect assessment of their resistance to high temperature compared with other bitumen aged at a traditionally adopted temperature of 163 °C.

Short-term aging RTFOT undoubtedly has disadvantages in the form of dedication to “hot” technology and difficulties in performing the test for hard or very highly modified bitumen [26]; however, this test allows the obtainment of samples for further tests that can verify the binders in terms of resistance to technological aging of bitumen. Research has confirmed that the use of two different bitumen in asphalt mix with the same composition, produced under the same conditions, will lead to greater aging in asphalt mix with bitumen which showed greater changes in physicochemical parameters after the RTFOT test [27].

**Figure 6:**
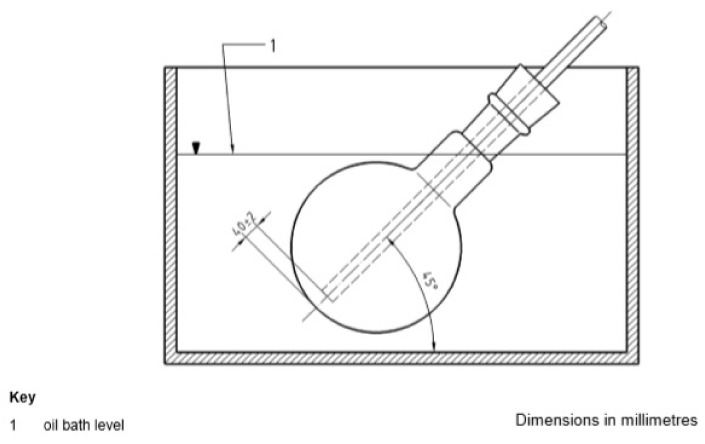
Schematic of RFT aging setup [EN 12607-3:2014].

Another standardized method is the RFT (*Rotating Flask Test*) bitumen aging simulation, initially described in the 1980s in the German standard DIN 52,016 [28]. The European standard EN 12607-3 [29] describing the test procedure was developed by the technical committee TC 336 in 2000. The method uses a rotary evaporator into which 100 g of bitumen sample is introduced. The conditions of the RFT aging procedure are 165 °C, time 150 min, and rotation speed of 20 rpm. In addition, air is supplied to the flask at a rate of 500 mL/min. Figure 6 shows a schematic of the RTF. An unquestionable advantage of the RFT method compared with RFTOT is the possibility of obtaining a single homogeneous sample of the aged binder for further tests, i.e., 100 g instead of 35 g from one container. Admittedly, as many as 8 containers can be used in the RFTOT test, but the removal of bitumen binder from these containers is problematic and if a sample larger than 35 g is required, the test material from at least two containers must be combined.

Other short-term bitumen aging methods have not been standardized, but only tested and developed by scientists. However, very good data validation with asphalt field measurements may result in changing the reference methods to one of the methods described below.

Due to the increasing popularity of long-term aging tests and the need to evaluate the binder during pavement operation, increasingly larger sample sizes are needed for testing after the technological aging tests. To obtain larger sample sizes (e.g., to simulate long-term aging), a preliminary method based on the RFT test was developed in the U.S.A. [30]. A 200 g sample is placed in a larger flask (1000 mL), and the test is carried out at 165 °C for 210 min. The airflow is set at 2000 mL/min and the rotation of the flask at 20 rpm. The modified RFT method achieves aging changes similar to the RTFOT method.

Another published modification of the RFT method [31] involves encasing the oil bath to stabilize the test temperature as well as to be able to use a higher temperature, e.g., 200 °C. Furthermore, the flask was placed in the evaporator at an angle of 45° to ensure that the entire sample volume was always below the oil level. The rotation speed was increased to 60 rpm and the airflow from 500 mL/min to 4000 mL/min. The modified RFT test using, for example, a test time of 85 min and a 50 g sample, allows for a similar aging effect as the RTFOT method, and after 60 min the effect is similar to TFOT. The equipment modified in this way provides greater possibilities for selecting different conditions for accelerated bitumen aging. Depending on the needs and asphalt mix production technology, the conditions of the RFT method can be selected in such a way as to correlate changes in bitumen during heating and contact with air.

Another proposed procedure mapping the technological aging of bitumen is the stirred airflow test (SAFT) method, which was supposed to solve the disadvantages associated with the aging of polymer-modified bitumen (insufficient flow in RTFOT containers, formation of a “crust” on the surface of the binder) [32]. The SAFT aging apparatus has a structure that is somewhat reminiscent of a bitumen oxidation reactor, as it contains a vessel in which the temperature of the medium is controlled, along with an air or nitrogen inlet and a stirrer. Comparative tests of the viscosity after SAFT versus RTFOT aging using the developed laboratory conditions performed both on paving grade and modified bitumen samples, revealed a satisfactory correlation [33].

Moreover, the advantage of the SAFT method over RTFOT results mainly from two aspects. Firstly, the time needed to perform the test is reduced to 45 min (including the 15 min needed to heat the sample to 160 °C), and secondly, the sample is always averaged due to its placement in one container and the use of stirring with the installed stirrer. The test temperature is the same as in the case of RTFOT, i.e., 163 °C, the mass of the sample subjected to aging is 250 g, the airflow during the aging period is set at 2000 mL/min, and the stirrer speed at 700 rpm. The aging cycle, where the bitumen is exposed to elevated temperature and oxygen in the air, is 30 min.

Despite solving the problematic technical aspects of the RTFOT method, the SAFT test was not generally accepted by industry and science. It was noted in [34] that the SAFT method, carried out using commercially available equipment, ages bitumen to a lower degree, especially in the case of high G* bitumen binders, hence the need to consider whether the procedure time should be extended from 30 to at least 35 min. In addition, a sample degassing step has been proposed, because air bubbles trapped in the bitumen affect the values of rheological properties measured. The studies conducted by the authors of the report revealed a worsened correlation of changes in the properties of bitumen after aging compared with the RTFOT method and bitumen recovered from short-term aged asphalt mix. Ultimately, the SAFT method was not recommended for introduction to the specification.

Table 1 summarizes the three most commonly used methods of short-term technological aging of bitumen, covered by the European standards of EN 12607-1, 2, and 3 series. Additionally, different aging conditions are included, provided in the standards used, among others, in North America, i.e., AASHTO and ASTM.

### 3.2. Long-Term Aging

Understanding the behavior of bitumen during technological aging prevents the use of binders with insufficient parameters for pavement construction, whereas to obtain a more complete characterization of changes in bitumen properties after several years of operation, additional tests should be performed. Long-term aging tests are most often performed on bitumen samples that have undergone a short-term aging cycle.

Pressure aging vessel (PAV), the procedure most widely used in the world for long-term aging tests, was developed under the SHRP program in the United States. The entire SHRP research program, completed in 1994 with published reports, was conducted for 5 years and had a budget of USD 150 million. This comprehensive research program was intended to improve the performance of asphalt and concrete products and to develop methods of highway maintenance as well as the issues of long-term durability of road surfaces [35]. Among the significant achievements of the program are the Superpave specifications for bitumen binders and asphalt mixes. The results of the Superpave program led to the publication in the asphalt industry, for the first time, of bitumen specification related to the functional properties AASHTO M320 [36], which was based on scientific knowledge, in particular the chemistry and mechanics of behavior of construction products such as bitumen binders.

**Figure 7:**
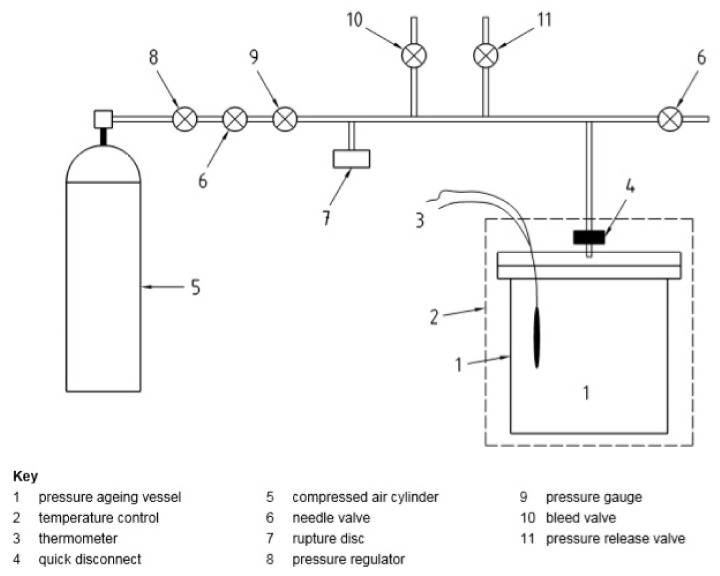
Diagram of the PAV aging setup [EN 14769-2012].

The accelerated long-term aging method in a PAV chamber is described in ASTM D6521 [37] and AASHTO R28 standards [38]. In Europe, however, the method was published as a standard EN 14769, issued in 2005. The procedure involves placing a 50 g sample of bitumen previously subjected to short-term aging in 140 mm diameter trays (TFOT trays can be used). Up to 10 trays can be placed in the PAV apparatus, so—if necessary—a total of about 500 g of asphalt can be simultaneously subjected to the procedure of accelerated long-term aging. Next, the air is supplied to the chamber of the PAV apparatus, which is pressurized to create an overpressure of 2.07 MPa. Figure 7 shows a schematic of the PAV. Aging is carried out for 20 h in the temperature range of 90–110 °C [39] depending on the grade of bitumen. The lower the penetration or the higher the upper performance grade temperature, the higher the aging temperature.

PAV provides a picture of changes taking place over a longer period of bitumen oxidation. Nevertheless, there are many objections to this aging method since the mechanism of accelerated hardening of the binder does not take into account all the key phenomena that take place during service life aging of pavement [40]. In particular, the high-pressure nature of aging is emphasized, which minimizes the evaporation of hydrocarbons from the binder sample, and the high-test temperature, which is unrealistic compared with the pavement temperature and, therefore, under elevated temperature conditions chemical changes in the bitumen which would not occur at lower temperatures may be preferred—this follows from the activation energy of many chemical reactions that can take place in the binder. Additionally, when testing polymer-modified bitumen, there is concern about the possibility of bitumen–polymer separation due to the static nature of the procedure (containers with bitumen do not rotate or spin).

To limit the influence of high temperature on the long-term aging of the bitumen, an alternative procedure called HIPAT (High Pressure Aging Test) based on the PAV method was developed in the United Kingdom [41]. The method uses a lower temperature of 85 °C and extends the aging time to 65 h. Although in the early phase of development the method was indicated as a tool for identifying binders with an increased potential for aging during use, at present, HIPAT is not often used in the asphalt industry. The provisions for a special long-term aging procedure at 85 °C are included in the European standard EN 14769 for PAV aging. Currently, only a few studies on the use of this aging method have continued [42], probably due to the time needed to carry out the research using this method.

The second main method used for accelerated aging to simulate long-term changes in bitumen is RCAT (Rotating Cylinder Aging Test). The method was developed in Europe at the Belgian Road Research Center [43] at the beginning of the 21st century. The method was standardized in 2007 in CE TC 336 and EN 15323. According to the procedure described in EN 15322, the RCAT test involves placing a sample weighing 525–550 g in a cylinder that has been preheated to the temperature at which the aging will take place. Bitumen is left at this temperature for about 60 min ± 5 min. During this time, oxygen is not fed to the cylinder and the cylinder is not rotated. After 60 min, in which the bitumen is considered to have reached the RCAT temperature (typically 90 °C), the cylinder is rotated at 1 rpm and oxygen is pumped to the cylinder at 4500 mL/min ± 500 mL/min. The pressure inside the cylinder is kept at 0.1 MPa. The first bitumen samples are collected after 17 and 65 h to monitor the hardening process. The sample in the RCAT method is aged for 140 ± 15 min.

**Figure 8:**
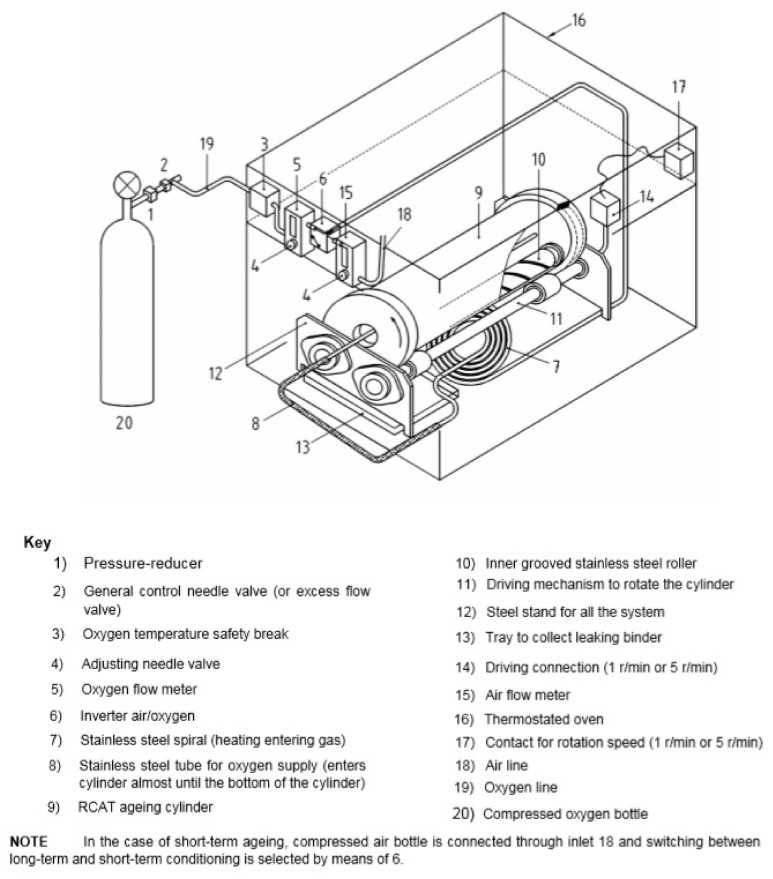
Schematic diagram of the RCAT aging apparatus (EN 15323:2017).

Moreover, using the RCAT apparatus and changing the aging conditions, an initial short-term aging cycle can be performed [44]; therefore, it is not necessary to conduct several RTFOT tests in order to obtain a sample mass of about 500 g for the long-term aging cycle using the RCAT method. In the technological aging cycle, oxygen is replaced with air at a fixed flow rate of 4000 mL/min ± 200 mL/min, the test temperature is 163 °C, and the aging time is 235 min ± 5 min. The cylinder is rotated five times faster than in the long-term aging test procedure. Figure 8 shows a schematic of the RCAT. After completion of the aging cycle, changes in the properties of the bitumen are determined both by empirical methods (penetration, softening point) and by FTIR spectroscopy identifying changes in the bitumen molecules, which prove that the bitumen aging extent is comparable to that carried out according to the RTFOT procedure.

Despite standardization, the European method is not as popular as the PAV method developed for the Superpave program and it is not included as an aging procedure in the specifications of bitumen binders. This is mainly due to the greater availability of PAV aging equipment and the longer test duration for RCAT. However, it should be emphasized that RCAT provides a unique opportunity to perform both short- and long-term aging.

A comparison of aging by PAV and RCAT methods shows that the values of bitumen tests made after long-term aging can be comparable. In particular, in the case of bitumen modified with SBS copolymer, when considering the softening point difference parameter, it was shown that PAV aging at 100 °C for 20 h, compared with the RCAT procedure at 90 °C for 140 h, causes a greater degradation of the polymer. Higher temperature and pressure are important in this case. On the other hand, the polymer-modified bitumen recovered from the exploited asphalt pavement did not show such hardening characteristics as would follow from the PAV procedure. In this case, the analysis of the molecular weight distribution using gel permeation/size exclusion chromatography (GPC/SEC) proves to be helpful in the interpretation of the results. The determinations carried out with GPC/SEC revealed that after 9 years of use of pavement asphalt, there was an even greater degradation of the polymer (decrease in molecular weight) and the compounds resulting from its degradation became a kind of plasticizer, preventing bitumen from strong hardening [45]. The usefulness of GPC/SEC for this type of investigation was described in detail in [46].

In Austria, a long-term simulation method called 3xRTFOT was used to specify paving grade bitumen characteristic. The requirement for the softening point after aging RTFOT and 3xRTFOT was defined as the maximum increase of 15 °C with respect to the softening point of the unaged binder. The 3xRTFOT involves performing 3 cycles according to the RTFOT methodology (3 *×* 75 min), leaving the conditions unchanged. The assessment of changes after long-term aging was introduced to more thoroughly verify the susceptibility of bitumens to overly quick aging. The 3xRTFOT method is sensitive to bitumen containing raw materials from visbreaking installations which, when used in the production of paving grade binders, can cause accelerated and significant adverse changes in asphalt pavement [47]. The process of visbreaking of heavy oil fractions (carried out at T: 455–510 °C, and P: 0.3–2 MPa) is considered a mild type of thermal cracking which became very popular in the 1980s. As a result of the conversion of the vacuum residue from the crude oil distillation, additional fuel fractions are obtained. Although the conversion with this technology amounts only to 10 to 25%, refineries which in those years had a visbreaking installation in their configuration achieved better financial ratios. Bitumen produced with the use of raw materials from the cracking process, e.g., made in a visbreaking installation, compared with binders produced from distillation fractions, show higher temperature sensitivity and lower resistance to oxidation and, consequently, more pronounced changes during use [48]. The source of accelerated aging is unsaturated hydrocarbons (olefins) and aromatic hydrocarbons, which are generated during thermal cracking at low pressure [49]. The research [50] compared the content of carbon and hydrogen in aromatic structures (H_arom._ And C_arom._) for the vacuum residue (feedstock) and the visbreaking residue (product) using nuclear magnetic resonance (NMR) spectroscopy. As can be seen from the data in Table 2, high molecular weight olefins behave very unstably in petroleum fractions, especially at elevated temperatures. Compounds from this group undergo secondary oxidation reactions, yielding a less useful raw material than the crude vacuum residue.

The unquestionable advantage of the 3xRTFOT method is the possibility of using the same research equipment for both technological and operational aging tests. However, taking into account the real temperature of pavement use compared with the aging process at 163 °C, different physical and chemical transformations take place than would be expected from a method simulating the long-term aging of bitumen.

A similar approach, the so-called ERTFOT163sra, was presented in South Africa [51], where the time of the RTFOT method to simulate the effect of long-term aging was extended to 325 min. In the meantime, at 205 min, a sample was taken to determine the trend of changes in the characteristics of the bitumen binder. The second version of alternatively modified RTFOT, ERTFOT100sra, additionally had the following parameters changed compared with the RTFOT method: the temperature was reduced to 100 °C and the procedure time was extended to 48 h. In addition, metal rods were introduced to aid the spinning of the bitumen layer as described in the fragment with the modified RTFOT method for short-term aging simulation. The study of the correlation of the ERTFOT163sra and ERTFOT100sra methods with PAV performed on bitumen from 7 sources, revealed that aging proceeds more gently at a lower temperature, despite the extended time. On the other hand, RTFOT extended to 325 min allows for obtaining changes in bitumen corresponding with the PAV method. It should be emphasized that the ERTFOT163sra method does not require a prior RTFOT test, as within 325 min there are aging changes taking into account both short- and long-term oxidative hardening of the bitumen.

Currently, the PAV method is often criticized. It has been demonstrated that it does not reflect changes taking place in asphalt mix during the service life of the road surface. Due to the complexity of the processes occurring in bitumen during pavement exploitation, it appears that none of the methods described above will fully reflect the phenomena taking place in reality [24]. At present, aging procedures are being developed that take into account, in addition to the effects of heat and oxygen as an oxidant, also the effect of UV, moisture and exhaust gases, especially in the case of bitumens used for the wear layer of asphalt mix. In addition, accelerated aging tests are sought to also take into account the characteristics of the filler used in the asphalt mix and its structure. For example, asphalt mix defined as porous asphalt contains a relatively large amount of void space, hence the oxidative aging of such a mix is increased compared with more densely packed asphalt mix, such as asphalt concrete.

To more adequately reflect the wear and tear of bitumen over 5 to 10 years, the Vienna University of Technology developed the VAPro (Viennese Aging Procedure) method [52], which uses conditions similar to the natural aging conditions occurring during their use. The method is an extension of the procedure proposed in the SHRP-A-383 report [53]. The temperature of the VAPro procedure is 60 °C, an overpressure of about 80 kPa, and the flow of air enriched with ozone and nitrogen oxides at the level of 0.9–1.1 L/min. Ozone and nitrogen oxides are produced using an ozone generator. The entire long-term aging procedure takes 3 days and is intended to reflect all changes occurring during the short- and long-term hardening of bitumen.

In the VAPro method, unlike the others described, the asphalt mix sample is aged, and then, following the aging cycle, the bitumen is recovered and the tests in parameters are performed for the recovered binder. On one hand, it is justified, as it takes into account the interaction of asphalt mix components and the type of asphalt mix; on the other hand, it complicates the preparation of the sample and its recovery after aging.

The test results for bitumens after VAPro aging compared with RTFOT and PAV aging differ significantly. Reported changes in binder properties are always greater after the VAPro procedure. In particular, the Austrian procedure provides greater changes than PAV, especially for asphalt mix samples from less typical sources, and better reflects the changes in the properties of low-temperature binders during operation. In the case of assessment of phase angle (δ) in the DSR, polymer-modified bitumens showed the expected change in δ after long-term aging (VAPro), while for the same samples after RTFOT and PAV procedures the changes were only slight [54].

A drawback of the VAPro procedure is the time necessary to prepare the sample because the sample is not only a binder, as in the methods described previously, but also a concentrated asphalt mix. As a result, the method takes into account the effect of mineral components and the packing density of the sample, but at the same time increases the requirements for the amount of material, time-consuming mixing of components, and compaction of asphalt mix. In addition, the time of the procedure is also 52 h longer than in the case of the PAV method.

Table 3 summarizes the most commonly used long-term (service life) aging methods, covered by European EN standards as well as AASHTO and ASTM standards used, among others, in North America.

This review did not cover test methods that take into account the role of UV and moisture in the aging of bitumen binders. These methods are of importance primarily for bitumen used for the wearing course of road surfaces.

## 4. Suggestions for the Future Work

This literature review, taking into account the industry standards used in laboratory practice in Europe and North America, and the authors’ experience in testing bitumen binders, allows us to propose the following changes with respect to the developed or currently modified methods of bitumen aging:

(a) For short-term aging, differentiation of the aging temperature or the aging time should be used to identify realistic changes to the bitumen during its processing to asphalt mix. Depending on the used temperature, i.e., hot, warm, semi-warm or cold technologies of asphalt mix select the appropriate bitumen aging temperature, i.e., when bitumen is used at 180 °C, this temperature should be used for the RTFOT procedure;

(b) The long-term aging of the bitumen, depending mainly on the chemical properties of the bitumen and climatic conditions, should also differ in relation to the location of the given bitumen in a specific layer of the pavement. Such differentiation would allow the elimination of the most susceptible bitumen from going into the wearing courses and, at the same time, if the criteria are met, enabling their use for the lower layers of asphalt pavements; (c) Further developments of bitumen aging demands tests that include the important role of factors such UV and moisture. Depending on bitumen origin and composition, aging behavior is expected to strongly differ. Thus, such tests could significantly contribute in the overall evaluation of aging processes.

The application of these proposals will have an impact on obtaining changes in bitumen similar to real ones, which is important both in the research aspect and in industrial application. Omitting these issues in product specifications or newly developed bitumen binder formulations in relation to the actual changes taking place in bitumen, when applying them to a given technology and end use, reduces the credibility of the results and conclusions.

## 5. Conclusions

The aging process of the bitumen binder in the road surface is very complex. It is affected by climatic conditions (temperature, especially extreme temperatures and the number of passes through 0 °C, air humidity, number of hours exposed to sun, precipitation, and air pollution), technological conditions (temperature and time of further processing) as well as conditions resulting from its chemical structure (oil source, production technology, additives and modifiers used). Therefore, it has not yet been possible to develop a method to completely simulate the actual bitumen aging, and this task seems difficult to implement at the moment. New developments in test methods that include the effect of UV light and moisture on bitumen aging are needed.

In laboratory practice, the most frequently used test procedures and equipment are in line with the RTFOT methodology in the case of short-term aging and the PAV method for the assessment of changes after long-term aging. This is mainly due to the imposition of these procedures in technical specifications, the availability of laboratory equipment, and the simplicity of laboratory procedures.

The changes recorded under the test conditions often do not correspond to the actual changes taking place during the processing of bitumen using the technology suitable for the production of the product for its intended final application.

An important issue, which is not fully understood with the currently used different bitumen application technologies, is the assumption of one aging temperature in RTFOT (most often it is 163 °C). In the case of very hard bitumen, e.g., 20/30, and on the other hand relatively soft bitumen 160/220, the use of the same laboratory aging temperature significantly differs from the temperatures used in the production technology of both hot mix asphalt and warm mix asphalt.

The same applies to the PAV procedure, although, compared with RTFOT, the choice is most often made between the temperatures of 80, 90, 100 or 110 °C. The selection is made based on inaccurate criteria.

Moreover, the simulation time of long-term aging is long, as far as performing the test is concerned. Performing the full bitumen aging cycle using the most popular procedures, such as RTFOT and PAV, requires nearly 24 h.

Undoubtedly, a fairly important aspect is the amount of material that is used in bitumen aging procedures. One aging cycle is often insufficient to fully characterize changes in bitumen properties or to prepare samples of special asphalt mixes. This leads to the need to prepare another series of samples and perform additional aging cycles.

A significant disadvantage of the bitumen aging procedures described in this article is the inability to record changes in bitumen parameters automatically during the aging simulation process. If a test sample has to be collected while the procedure is in progress, it is necessary to pause, draw samples manually, and restart aging to complete the cycle.

Arguably, the development of bitumen testing methods should focus on the automation of procedures in which parameters can be dynamically changed with simultaneous recording of the sample properties.

New methods of simulation of aging focus on the need to eliminate the above-mentioned inconveniences, but none of the methods described so far has been accepted by scientists or industry.

## Figures and Tables

**Table 1:** Conditions for short-term aging of bitumen using the three methods described in European standards.

Method	Aging Time (min)	Sample Mass (g)	Air Flow (mL/min)	Temperature (°C)	Remarks
TFOT	300	50	Without forced circulation	163	Currently used for soft paving grade bitumen, i.e., V6000 acc. To EN 12591
RTFOT	75	35 (8 × 35 g = 280 g)	4000	163	According to AASHTO T240, aging time is 85 min
RFT	150	100	500	165	

**Table 2:** Measurement of H_arom._ and C_arom._ using NMR of residues from Iranian heavy crude oil and Kirkuk crude oil [50].

Parameter	Iranian Heavy Crude Oil		Kirkuk Crude Oil	
	Vacuum Residue	Visbreaking Residue	Vacuum Residue	Visbreaking Residue
H_arom._	5.40	7.50 (+ 38.8%)	5.20	7.90 (+ 51.9%)
C_arom._	25.00	31.00 (+ 24%)	23.50	31.00 (+ 31.9%)

**Table 3:** Long-term aging conditions for basic methods standardized in EN, AASHTO and ASTM standards.

Method	Aging Time (h)	Sample Mass (g)	Gas Flow Rate (mL/min)	Temperature (°C)	Pressure (mPa)	Remarks
PAV	20	500 (10 × 50 g)	Air is used without forced flow	90–110 °C	2.07	110 °C is used to simulate, e.g., desert conditions
RCAT	140	525–550	Oxygen 4500 mL/min	Typical 90 °C, max 100 °C	0.1	Cylinder rotations 1 rpm
PAV (HIPAT)	65	500–50 (10 × 50 g)	Air is used without forced flow	85 °C	2.07	If 85 °C is used, the time should be extended to 65 h.

## Data Availability

Not applicable.
